# Recognition of Self and Viral Ligands by NK Cell Receptors

**DOI:** 10.1111/imr.13435

**Published:** 2025-01-02

**Authors:** Roy A. Mariuzza, Pragya Singh, Sharanbasappa S. Karade, Salman Shahid, Vijay Kumar Sharma

**Affiliations:** ^1^ W. M. Keck Laboratory for Structural Biology University of Maryland Institute for Bioscience and Biotechnology Research Rockville Maryland USA; ^2^ Department of Cell Biology and Molecular Genetics University of Maryland College Park Maryland USA; ^3^ College of Natural and Mathematical Sciences University of Maryland College Park Maryland USA

**Keywords:** KIR, Ly49, MHC, NK receptor, structure, virus

## Abstract

Natural killer (NK) cells are essential elements of the innate immune response against tumors and viral infections. NK cell activation is governed by NK cell receptors that recognize both cellular (self) and viral (non‐self) ligands, including MHC, MHC‐related, and non‐MHC molecules. These diverse receptors belong to two distinct structural families, the C‐type lectin superfamily and the immunoglobulin superfamily. NK receptors include Ly49s, KIRs, LILRs, and NKG2A/CD94, which bind MHC class I (MHC‐I) molecules, and NKG2D, which binds MHC‐I paralogs such MICA and ULBP. Other NK receptors recognize tumor‐associated antigens (NKp30, NKp44, NKp46), cell–cell adhesion proteins (KLRG1, CD96), or genetically coupled C‐type lectin‐like ligands (NKp65, NKR‐P1). Additionally, cytomegaloviruses have evolved various immunoevasins, such as m157, m12, and UL18, which bind NK receptors and act as decoys to enable virus‐infected cells to escape NK cell‐mediated lysis. We review the remarkable progress made in the past 25 years in determining structures of representatives of most known NK receptors bound to MHC, MHC‐like, and non‐MHC ligands. Together, these structures reveal the multiplicity of solutions NK receptors have developed to recognize these molecules, and thereby mediate crucial interactions for regulating NK cytolytic activity by self and viral ligands.

## 1 Introduction

Natural killer (NK) cells are innate immune lymphocytes that recognize and kill tumor and virally infected cell [1, 2, 3, 4]. The cytotoxic activity of NK cells is controlled by positive signaling activating receptors (resulting in target cell lysis) and negative signaling inhibitory receptors (preventing lysis) [5, 6]. The dynamic interplay between these signals determines the outcome of NK cell encounters with target cells. The dominant signal transmitted to a NK cell is inhibitory and arises from the interaction of its inhibitory receptors with normal levels of MHC class I (MHC‐I) on healthy cells. If MHC‐I expression is reduced by tumorigenic or infectious processes, the inhibitory signal is attenuated and the NK cell is activated. The process through which NK cell receptors direct the cytotoxic activity of NK cells against tumor or virally infected cells with reduced MHC‐I expression is termed “missing‐self” recognition.

NK receptors belong to two structural families: the C‐type lectin superfamily and the immunoglobulin (Ig) superfamily [7]. Both superfamilies include inhibitory and activating receptors. Besides MHC molecules, NK receptors recognize MHC‐related and non‐MHC ligands of both cellular and viral origins. Inhibitory receptors responsible for monitoring MHC‐I expression include C‐type lectin‐like Ly49 receptors in rodents and killer Ig‐like receptors (KIRs) and leukocyte Ig‐like receptors (LILRs) in humans. In addition to attenuated inhibitory signals, activating signals are required to trigger NK cells [5, 6]. Activating signals are delivered by a variety of NK receptors, among them NKG2D, NKR‐P1, NKp30, NKp44, NKp46, and NKp65, whose ligands include both MHC‐related and non‐MHC molecules. Viral immunoevasins such as cytomegalovirus m157 and UL18 that bind NK receptors have also been identified.

Remarkable progress has been made over the last 25 years in determining crystal structures of representative examples of most known NK receptors, in both free form and bound to MHC, MHC‐like, and non‐MHC ligands (Table 1). These include both C‐type lectin‐like (Ly49s, NKG2D, NKG2A/CD94, NKp65, KLRG1, NKR‐P1) and Ig‐like (KIRs, LILRs, NKp30, NKp44, NKp46, CD96, 2B4, TIGIT) receptors. Collectively, these structures, which we review here, have revealed the diverse solutions that NK receptors have evolved to recognize cellular and viral ligands, and thereby regulate NK cell cytotoxicity.

**TABLE 1:** Structures of NK cell receptors and complexes.

NK receptor or complex	PDB code (references)	NK receptor or complex	PDB code (references)
Ly49C	3C8J [8]	LILRB1–HLA‐A2	1P7Q [9]
Ly49G	3CAD [8]	LILRB1–UL18	3D2U [10]
Ly49I	1JA3 [11]	LILRB2–HLA‐G	2DYP [12]
Ly49L	3G8L [13]	NKp30	3NOI [14]
Ly49A–H‐2D^d^	1QO3 [15]	NKp44	1HKF [16]
Ly49C–H‐2K^b^	1P4L [17]	NKp46	1P6F [18]
Ly49C–H2‐Q10	5J6G [19]	NKp30–B7‐H6	3PV6 [20]
Ly49H–m157	4JO8 [21]	NKG2D	1MPU [22]
KIR2DL1	1NKR [23]	NKG2D–MICA	1HYR [24]
KIR2DS2	1M4K [25]	NKG2D–RAE‐1β	1JSK [26]
KIR2DL2	2DL2 [27]	NKG2D–ULBP3	1KCG [28]
KIR2DS4	3H8N [29]	NKG2A/CD94–HLA‐E	3CDG [30]
KIR2DL3	1B6U [31]	NKG2D–ULBP6	4S0U [32]
KIR2DL4	3WYR [33]	KLRG1	3FF9 [34]
KIR2DL2–HLA‐Cw3	1EFX [35]	KLRG1–E‐cadherin	3FF8 [34]
KIR2DL1–HLA‐Cw4	1IM9 [36]	CD96–necl‐2	6ARQ [37]
KIR3DL1–HLA‐B*57:01	3VH8 [38]	NKp65–KACL	4IOP [39]
KIR2DL3–HLA‐C*07:02	6PAG [40]	NKR‐P1A–LLT1	5MGT [41]
KIR2DL2–HLA‐C*07:02	6PA1 [40]	NKR‐P1B–Clr‐b	6E7D [42]
KIR3DL1–HLA‐A*24:02	7K80 [43]	NKR‐P1B–m12	5TZN [44]

## 2 Recognition of MHC‐I by LY49 Receptors

The highly polymorphic Ly49 receptors, constituting a family of at least 23 members in mice (Ly49A–W), are the primary MHC‐monitoring molecules on rodent cells [45, 46]. Most Ly49s inhibit NK cell‐mediated cytolysis upon binding one or more H‐2D or H‐2K ligands, but some Ly49s are activating [45, 47, 48]. Although Ly49s generally recognize MHC‐I independently of the MHC‐bound peptide, some Ly49s, notably Ly49C and Ly49I, possess considerable peptide selectivity [49, 50]. Ly49s belong to the C‐type lectin family of proteins but lack a functional calcium‐binding site [7]. Ly49s are homodimeric type II transmembrane proteins, with each chain containing a C‐type lectin‐like domain named the natural killer receptor domain (NKD). The NKDs of the Ly49 homodimer each are connected to the transmembrane and cytoplasmic regions by very long stalks of ~70 amino acids.

Crystal structures have been reported for several Ly49 receptors in unbound form (Ly49C, Ly49G, Ly49I, and Ly49L) [8, 11, 13], as well as for Ly49A bound to H‐2D^d^ [15], Ly49C bound to H‐2K^b^ [8, 17], Ly49C bound to the non‐classical MHC‐Ib molecule H2‐Q10 [19], and Ly49H bound to the mouse cytomegalovirus (MCMV) immunoevasin m157 [21]. (Table 1). Together, these structures have revealed the molecular basis for MHC‐I recognition by Ly49s, the mechanism of MHC‐I engagement in *cis* and *trans*, and the strategy viral immunoevasins employ to target Ly49s.

**FIGURE 1:**
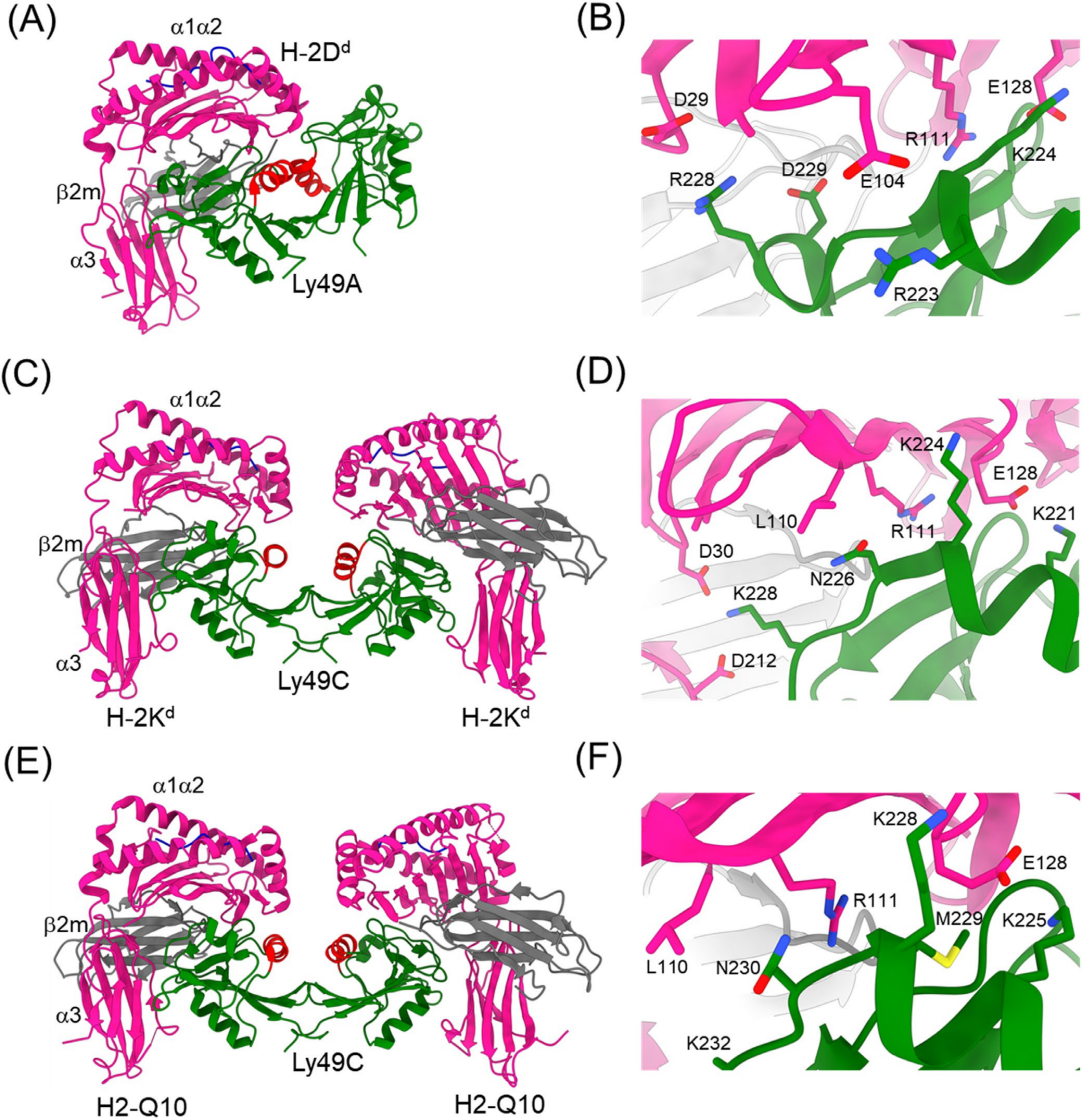
Structures of Ly49–MHC complexes. (A) Ly49A bound to H‐2D^d^ (PDB accession code 1QO3) [15]. The α1, α2, and α3 domains of the MHC‐I heavy chain are deep pink; β_2_m is gray; the MHC‐bound peptide is blue; the Ly49A dimer is green; the α2 helices of the Ly49A dimer are red. The complex is oriented with the NK cell at the top and the target cell at the bottom. (B) The Ly49A–H‐2D^d^ interface. The side chains of contacting residues are drawn in stick representation. (C) Structure of Ly49C bound to H‐2K^b^ (1P4L) [17]. (D) The Ly49C–H‐2K^b^ interface. (E) Structure of Ly49C in complex with H2‐Q10 (5J6G) [19]. (F) The Ly49C–H2‐Q10 interface.

The Ly49 NKD monomer comprises two α‐helices (α1 and α2) and two antiparallel β‐sheets consisting of seven β‐strands (Figure 1A,C,E). On the NK cell surface, Ly49s exist as dimers that may adopt two distinct conformations: “closed” and “open.” The closed conformation is exemplified by Ly49A, in which the C‐terminal ends of the α2 helices are juxtaposed (Figure 1A). The open conformation is exemplified by Ly49C, in which the α2 helices do not make contact (Figure 1C,E). In the Ly49A–H‐2D^d^ complex (Figure 1A), the Ly49A homodimer engages a single H‐2D^d^ molecule at a site underneath the peptide‐binding platform of MHC‐I using only one of its subunits [15]. This site partially overlaps the binding site for CD8. It is formed by the α1/α2, α3 and β_2_‐microglobulin (β_2_m) domains of H‐2D^d^. In contrast, the Ly49C dimer in the Ly49C–H‐2K^b^ complex [17] binds H‐2K^b^ bivalently, such that each subunit engages MHC‐I at a site equivalent to the Ly49A binding site on H‐2D^d^ (Figure 1C). Ly49C interacts similarly with H2‐Q10 (Figure 1E) [19]. This MHC‐Ib molecule may contribute to regulating NK cell responses during cellular stress.

The different dimerization geometries of Ly49A and Ly49C explain the different modes of MHC engagement by these NK receptors. The closed Ly49A dimer cannot engage two MHC‐I molecules simultaneously, in the manner of the open Ly49C dimer, because this would produce major steric clashes between the MHC ligands. However, NMR analysis demonstrated that Ly49A adopts predominantly the open conformation in solution that can bind two MHC‐I molecules [51], suggesting that Ly49s probably exist on the NK cell surface in dynamic equilibrium between closed and open forms.

Ly49s display specificity for different MHC alleles. Thus, whereas Ly49A only binds H‐2D^d^ and H‐2D^k^, the less specific Ly49C recognizes H‐2K^b^, H‐2K^d^, H‐2D^b^, H‐2D^d^, and H‐2D^k^ [46, 50]. Specificity is controlled mainly by residues 218–231, which exhibit high sequence variability across the Ly49 family [8]. This region adopts markedly different conformations in Ly49A (Figure 1B) and Ly49C (Figure 1D). It divides Ly49s that recognize both H‐2D and H‐2K alleles from those that recognize only H‐2D. Interactions at the Ly49C–H2‐Q10 interface (Figure 1F) resemble those in the Ly49C–H‐2K^b^ interface (Figure 1D), in agreement with the conserved nature of these MHC ligands [19].

The absence of direct contacts between the MHC‐bound peptide and Ly49A in the Ly49A–H‐2D^d^ complex explains why MHC‐I recognition by Ly49A is independent of peptide sequence in both binding and cellular assays [15] (Figure 1A). In contrast, Ly49C exhibits surprising peptide selectivity, despite the total absence of direct contacts between Ly49C and peptide in the Ly49C–H‐2K^b^ complex [8, 17] (Figure 1C). Molecular dynamics simulations support a role for peptide‐dependent dynamic tuning of electrostatic interactions across the Ly49C–H‐2K^b^ interface in peptide sensitivity [52]. According to this dynamic allostery model, different peptides alter the flexibility of H‐2K^b^, which in turn affects the strength of electrostatic interactions with Ly49C. However, the biological role of the peptide selectivity of certain Ly49s, like that of KIRs (see below), remains to be elucidated.

Ly49 receptors have been shown to bind MHC‐I molecules expressed on both target cells (in *trans*) and on the same NK cell (in *cis*) [53, 54]. Several other cell surface receptors, including LILR/PIR‐B, siglec‐2, and Notch receptors, also interact with ligands on opposing cells, as well as on the same cell [55, 56]. *Cis* interactions function to modulate (decrease or increase) the threshold at which a biological response is generated. In the case of Ly49s, *cis* interactions between Ly49s and MHC‐I promote NK cell activation by rendering Ly49s unavailable for *trans* interactions, thereby lowering the threshold at which NK cell activation surpasses inhibition [56]. In addition, *cis* interactions are required for NK cell education [57]. In order to engage MHC‐I in *cis* versus *trans*, Ly49s must drastically reorient their NKDs relative to the NK cell membrane. The long stalk region of Ly49s (~70 residues), which comprises three α‐helical segments linking the NKD to the transmembrane region, provides the necessary flexibility [13].

## 3 LY49 Recognition of A Viral Immunoevasin

**FIGURE 2:**
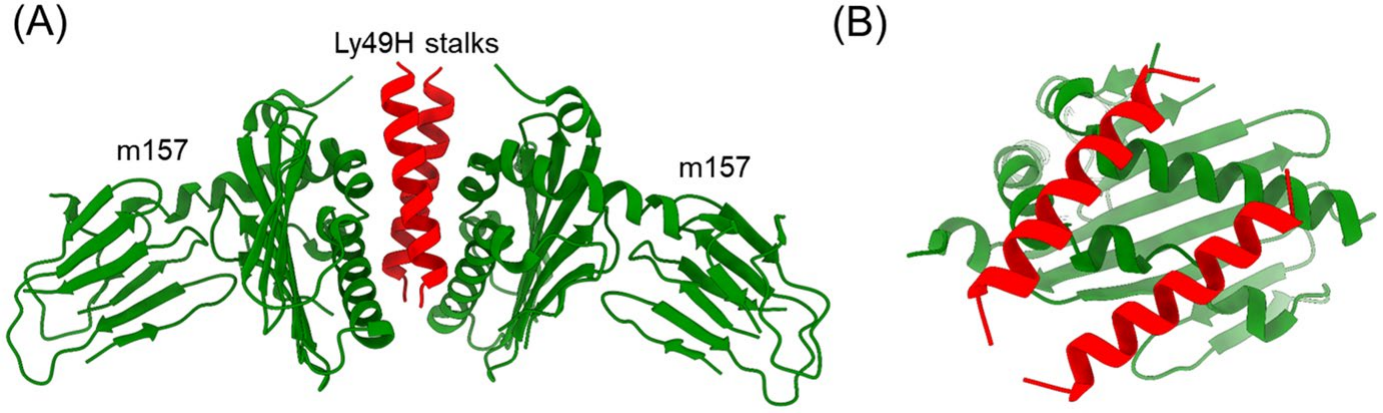
Structure of the MCMV immunoevasin m157 in complex with Ly49H stalks. (A) Side view of the Ly49H–m157 complex (4JO8) [21]. Two m157 monomers (green) bind the α‐helical Ly49H stalks (red). (B) Top view of the Ly49H–m157 complex. The Ly49H stalks straddle the α1/α2 platform of m157.

Studies of the susceptibility of different mouse strains to infection by mouse cytomegalovirus (MCMV) have shown that NK cells can directly interact with certain viral pathogens [58]. Resistance to MCMV infection in C57BL/6 mice is mediated by Ly49H, an activating receptor, which impairs viral replication [59, 60, 61, 62, 63]. BALB/c mice, in contrast, lack Ly49H and are susceptible to MCMV infection. Ly49L binds directly to m157, a viral glycoprotein and MHC‐I homolog which is expressed on MCMV‐infected cells [64, 65]. A crystal structure of the Ly49H–m157 complex revealed that m157 binds to the stalk region of Ly49H and not to the NKDs [21] (Figure 2A). Two m157 monomers engage the Ly49H dimer such that the stalks straddle the α1/α2 platform of m157 (Figure 2B). The specificity of m157 for different Ly49 family members is dictated by sequence differences in the stalk region.

## 4 Recognition of MHC‐I by KIR Receptors

The highly polymorphic KIR family of NK receptors contains the principal MHC‐monitoring molecules on primate NK cells and includes both activating and inhibitory receptors. In sharp contrast to Ly49s, which are type II transmembrane proteins composed of C‐type lectin‐like domains, KIRs are type I transmembrane proteins composed of two (D1 and D2; termed KIR2D) or three (D0, D1, and D2; termed KIR3D) extracellular C2‐type Ig‐like domains [7]. KIR2D receptors bind HLA‐C alleles while KIR3D receptors recognize HLA‐A and HLA‐B alleles. The structural basis for recognition of HLA‐C by two‐domain KIR2D receptors has been elucidated by crystallographic analysis of KIR2D molecules in free form [23, 25, 27, 29, 31] and bound to HLA‐Cw3, HLA‐Cw4, and HLA‐C*07:02 (Table 1) [35, 36, 40]. The way three‐domain KIR3D receptors bind HLA‐A and HLA‐B was revealed by structures of KIR3D molecules in complex with HLA‐A*24:02 and HLA‐B*07:02 [38, 43].

**FIGURE 3:**
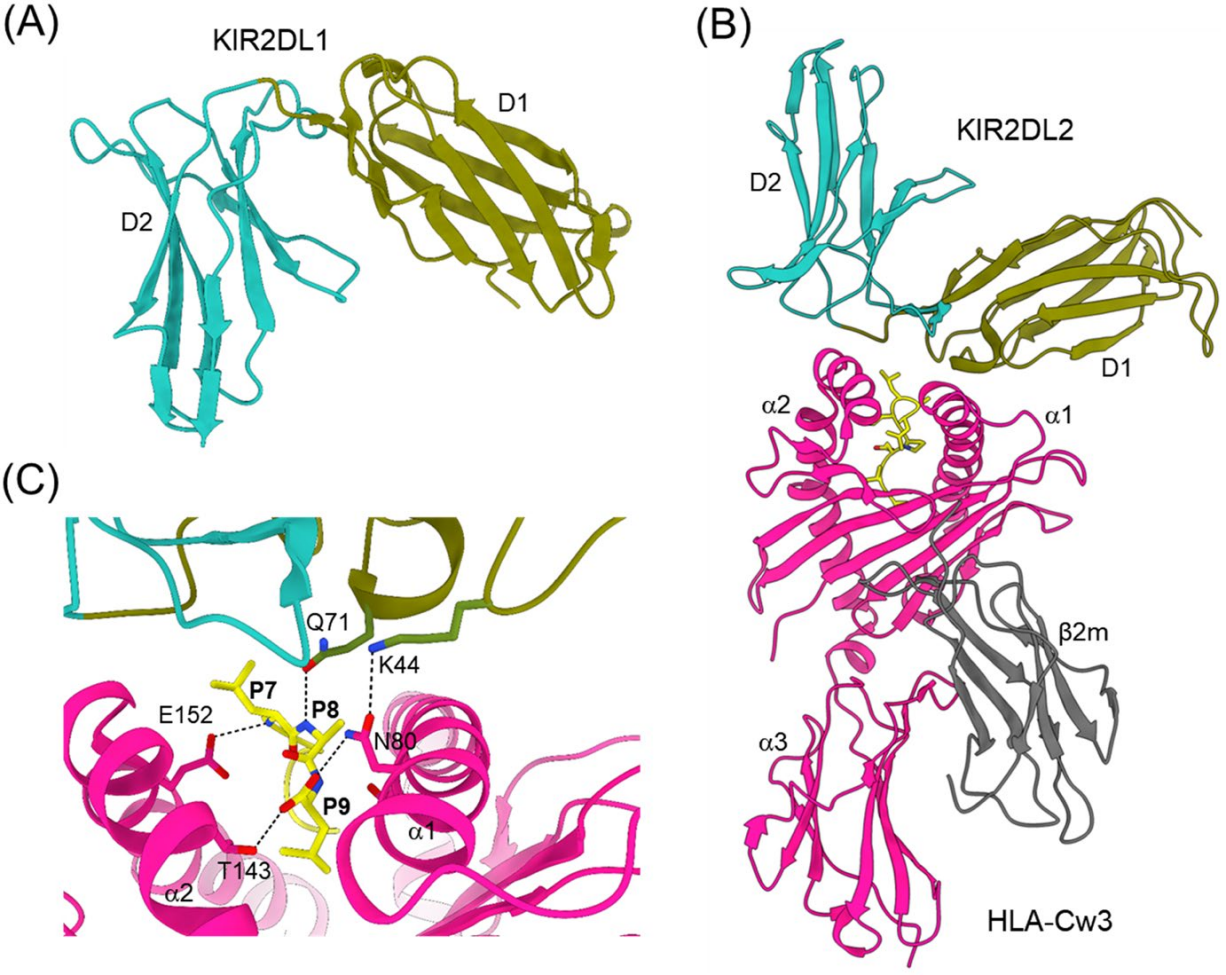
Structures of KIR2DL and a KIR2DL–HLA‐C complex. (A) Structure of unbound KIRDL1 (1NKR) [23], which comprises Ig‐like D1 and D2 domains. (B) Structure of KIR2DL2 bound to HLA‐Cw3 (1EFX) [35]. The α1, α2, and α3 domains of the MHC‐I heavy chain are deep pink; β_2_m is gray; the MHC‐bound peptide is yellow. (C) Close‐up of the KIR2DL2–HLA‐Cw3 interface. Hydrogen bonds are drawn as black dotted lines.

The tandem Ig‐like D1 and D2 domains of KIR2D receptors are linked by a short hinge of three to five residues (Figure 3A). In both the KIR2DL2–HLA‐Cw3 [35] and KIR2DL1–HLA‐Cw4 [36] complexes, the KIRs contact the α1 and α2 helices of HLA‐C and the C‐terminal portion of the MHC‐bound peptide, such that the D1–D2 axis is orthogonal to the peptide (Figure 3B). This docking mode is completely different from that of Ly49 NK receptors (Figure 1A,C) and roughly resembles the way T cell receptors bind MHC. The KIR2DL2–HLA‐Cw3 [35] and KIR2DL1–HLA‐Cw4 [36] structures explain the allelic specificity of KIR2DL receptors. With the sole exception of Asn80, all 12 HLA‐Cw3 residues that contact KIR2DL2 are conserved in all HLA‐C alleles. On the receptor side of the interface, all 16 KIR2DL2 residues that contact HLA‐HLA‐Cw3 are conserved in KIR2DL1, except Lys44 and Met70. In the KIR2DL2–HLA‐Cw3 structure, KIR2DL2 Lys44 forms a hydrogen bond with HLA‐Cw3 Asn80 that cannot be formed with KIR2DL1 Met44 (Figure 3C). In the KIR2DL1–HLA‐Cw4 complex, HLA‐Cw4 Lys80 is located in a negatively charged pocket of KIR2DL1 where it contacts Met44. If Me44 were replaced by Lys44, as in KIR2DL2, the resulting charge repulsion with HLA‐Cw4 Lys44 would abrogate binding.

As observed for Ly49 receptors [49, 50, 52], the binding of some KIRs to MHC‐I is peptide‐dependent. For example, the activating receptor KIR2DS4 exhibits a high degree of peptide specificity that includes an epitope conserved in bacteria [66]. Recently, a systematic screen was performed, totaling more than 3500 interactions, to determine the specificity of KIR2D receptors for peptides presented by HLA‐C ligands [67]. Whereas KIR2DL1 was agnostic about peptide sequence, KIR2DL2, KIR2DL3, and KIR2DS1 were highly peptide specific. This suggests that NK cell responses can be shaped by HLA‐I‐bound immunopeptidomes, which may help explain some of the numerous disease associations with specific combinations of KIRs and HLA‐I allotypes. Contacts between KIRs and MHC‐bound peptides observed in crystal structures of KIR2D–HLA‐C complexes [35, 36] provide a molecular basis for preferential peptide recognition.

**FIGURE 4:**
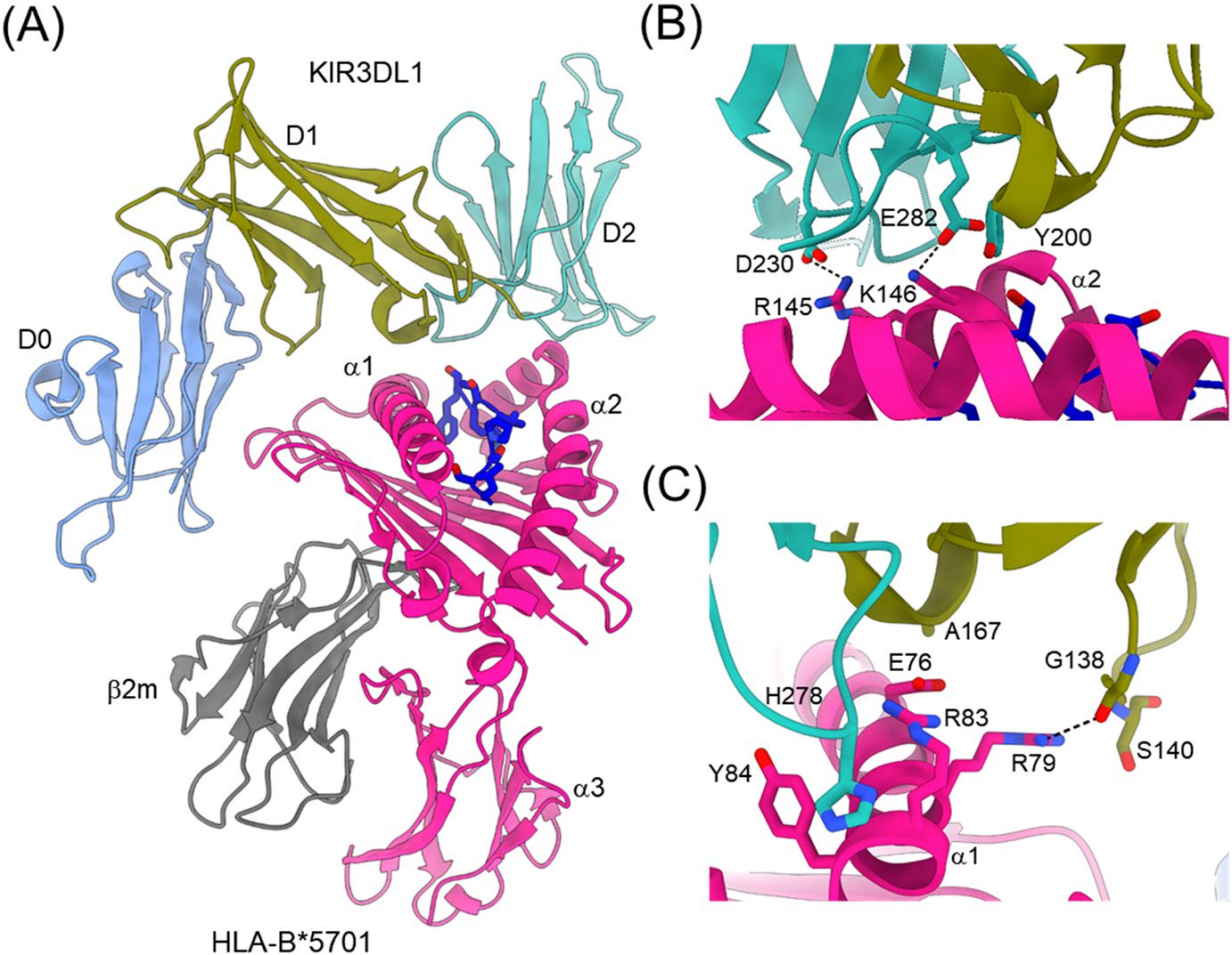
Structure of KIR3DL1 bound to HLA‐B*5701. (A) Structure of the KIR3DL1–HLA‐B*5701 complex (3VH8) [38]. The α1, α2, and α3 domains of the MHC‐I heavy chain are deep pink; β_2_m is gray; the MHC‐bound peptide is dark blue. (B) Contacts between KIR3DL1 and the α2 helix of HLA‐B*5701. Dotted black lines represent hydrogen bonds. (C) Contacts between KIR3DL1 and the α1 helix of HLA‐B*5701.

In the complex between KIR3DL1 and HLA‐B*5701 [38], KIR3DL1 binds MHC‐I with its D1 and D2 domains located over the C‐terminal half of the peptide‐binding groove (Figure 4A). This orientation resembles that of the KIR2DL2–HLA‐Cw3 complex (Figure 3B). KIR3DL1 displays an extended conformation that permits D0 to extend toward β_2_m and contact a region on HLA‐B*5701 that is highly conserved across HLA‐A and HLA‐B molecules. D1 interacts with the α1 helix and self‐peptide. D2 engages the α2 helix at residues 142–151, which exhibit limited polymorphism across HLA‐B alleles (Figure 4B). KIR3DL1 recognizes HLA allotypes containing the Bw4 epitope, which comprises amino acids 77–83 on the α1 helix. Contacts between D1 of KIR3DL1 and the Bw4 epitope (Figure 4C) explain the allelic specificity of KIR3DL receptors [38].

The structure of KIR3DL1 bound to HLA‐A*24:02 [43] showed that recognition of HLA‐A*24:02 by KIR3DL1 uses identical contacts as the HLA‐B*5701 ligand. Namely, D1 and D2 are positioned over the α1 and α2 helices, respectively, of the HLA‐A*24:02 peptide‐binding cleft, while D0 contacts the side of the MHC‐I molecule. However, functional analyses showed that KIR3DL1 recognition of HLA‐A*24:02 is more sensitive to substitutions in the α2 helix than is recognition of HLA‐B*5701, thereby establishing a ligand hierarchy for this KIR receptor.

## 5 LILR Recognition of MHC‐I and A Viral Immunoevasin

The human LILR family of immunoreceptors is expressed on NK cells, B cells, T cells, and dendritic cells [68]. The mouse orthologs of LILRs are called paired immunoglobulin receptors (PIRs). Similar to KIRs, LILRs possess either two or four tandem extracellular Ig‐like domains. LILRA1, LILRA2, LILRA3, LILRB1, and LILRB2 bind HLA‐A, ‐B and ‐C. However, several other LILRs (LILRA4, LILRA5, LILRA6, LILRB3, and LILRB4) do not seem to recognize MHC‐I molecules. In addition to detecting MHC‐I, LILRs contribute to immune responses to viral infections. Crystal structures have been determined of LILRB1 in unbound form [69] and in complex with HLA‐A2 [9] and UL18, a MHC‐I mimic encoded by human cytomegalovirus (HCMV) (Table 1) [10]. Structures have also been reported for LILRB2 in unliganded form [70] and bound to HLA‐G [12].

**FIGURE 5:**
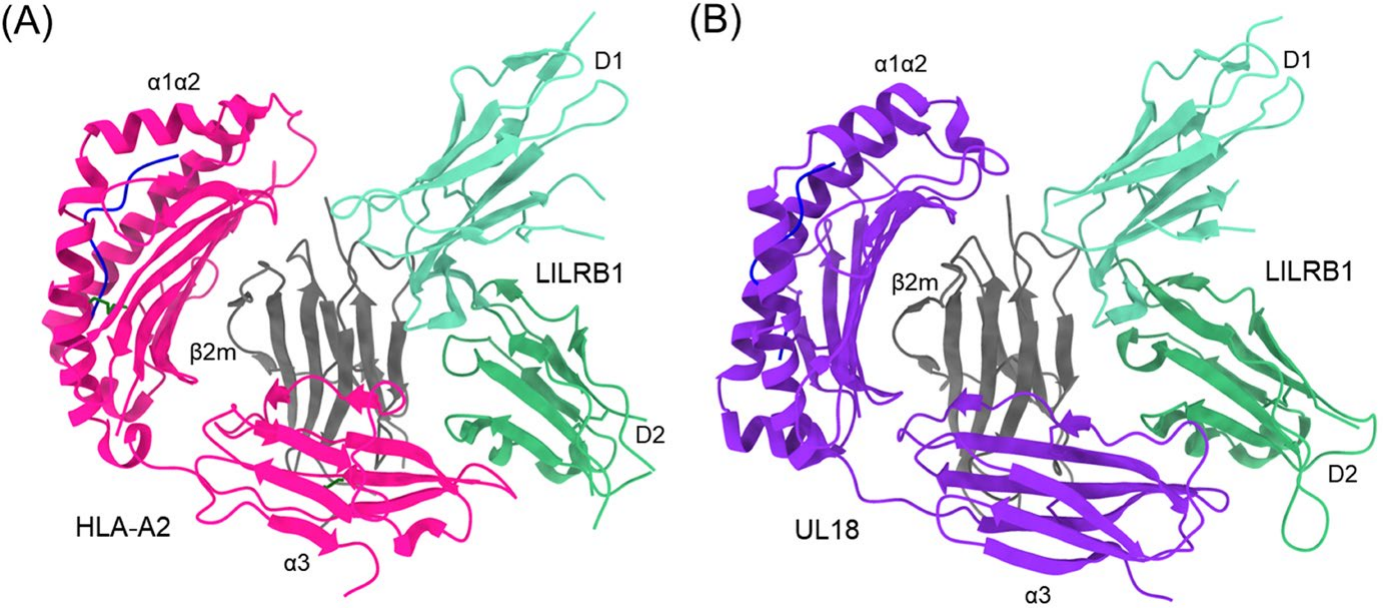
Interaction of LILRB1 with HLA‐A2 and the HCMV immunoevasin UL18. (A) Structure of LILRB1 in complex with HLA‐A2 (1P7Q) [9]. The α1, α2, and α3 domains of the MHC‐I heavy chain are deep pink; β_2_m is gray; the MHC‐bound peptide is blue. (B) Structure of LILRB1 bound to the HCMV MHC‐I mimic UL18 (3D2U) [10]. The α1, α2, and α3 domains of UL18 are violet; β_2_m is gray; the UL18‐bound peptide is blue.

Similar to KIR2D (Figure 3A), the two tandem Ig‐like domains (D1 and D2) of both LILRB1 and LILRB2 form a bent structure (Figure 5A) [9]. LILRB1 D1D2 contacts the relatively nonpolymorhic HLA‐A2 α3 domain and the invariant β_2_m subunit, as well as the D1–D2 interdomain hinge region. LILRB2 recognizes the HLA‐G α3 domain and β_2_m in a similar manner [12]. This binding mode differs completely from that of KIRs (Figure 3B). The focus of LILRs on conserved regions of MHC‐I explains their broad recognition of numerous HLA alleles independently of peptide.

Cytomegaloviruses encode proteins that impair recognition by both NK cells and T cells and that interfere with antigen processing and presentation [71, 72, 73]. Several of these immunoevasins are structural homologs of MHC‐I molecules. UL18 is an MHC‐I mimic encoded by HCMV that enables HCMV‐infected cells to escape NK cell‐mediated lysis by binding to the inhibitory receptor LRLB1 [74, 75]. UL18 binds LILRB1 > 1000‐fold more tightly than MHC‐I, such that UL18 competes effectively with MHC‐I for binding to LILRB1 [76].

The structure of the UL18–LILRB1 complex is strikingly similar to those of the LILRB1–HLA‐A2 and LILRB2–HLA‐G complexes [10], even though UL18 shares only ~25% sequence with its MHC‐I counterparts. The LILRB1 D1 domain contacts the UL18 α3 domain while the D1–D2 interdomain hinge contacts β_2_m (Figure 5B). No variable residues in the UL18 α1 domain contact LILRB1. Better surface complementarity and additional salt bridges in the LILRB1–UL18 interface compared with the LILRB1–HLA‐A2 interface are the likely reason for the > 1000‐fold higher affinity of UL18.

## 6 Natural Cytotoxicity Receptors

The natural cytotoxicity receptor (NCR) family in humans comprises NKp30 (CD337), NKp44 (CD336), and NKp46 (CD335) [77]. These potent activating receptors are type I membrane glycoproteins containing one (NKp30, NKp46) or two (NKp44) extracellular Ig‐like domains [78]. NCRs have a major role in enabling NK cells to lyse diverse cancer cells, including leukemias, neuroblastomas, and carcinomas [77, 78]. They are also involved in immune responses against multiple viruses, including influenza, hepatitis C, and West Nile virus [79, 80, 81, 82].

Ligands for NCRs have proven elusive, but significant progress in identifying them has been made. NKp44 and NKp46 bind influenza and other viral hemagglutinins via recognition by the hemagglutinin of sialic acid moieties on *N*‐linked glycans of these NCRs [79, 83]. The extracellular matrix protein nidogen‐1 (NID1) has also been reported as an NKp44 ligand [84], as has a novel isoform of mix‐lineage leukemia‐5 protein (MLL5) that is expressed on tumor cells but not normal cells [85]. Complement factor P was identified as a ligand for NKp46 [86]. NKp46 also recognizes externalized calreticulin, which translocates from the ER to the cell membrane during ER stress [87]. NKp30 recognizes the nuclear factor BAT3 [88], the tegument pp65 protein of HCMV [89], and β‐1,3‐glucan, a component of fungal cell walls [90]. NKp30 also recognizes the cell surface protein B7‐H6, a member of the B7 family that is selectively expressed on a wide variety of tumors [91, 92, 93]. The interaction of NKp30 with B7‐H6 results in interferon‐γ production and tumor cell killing.

**FIGURE 6:**
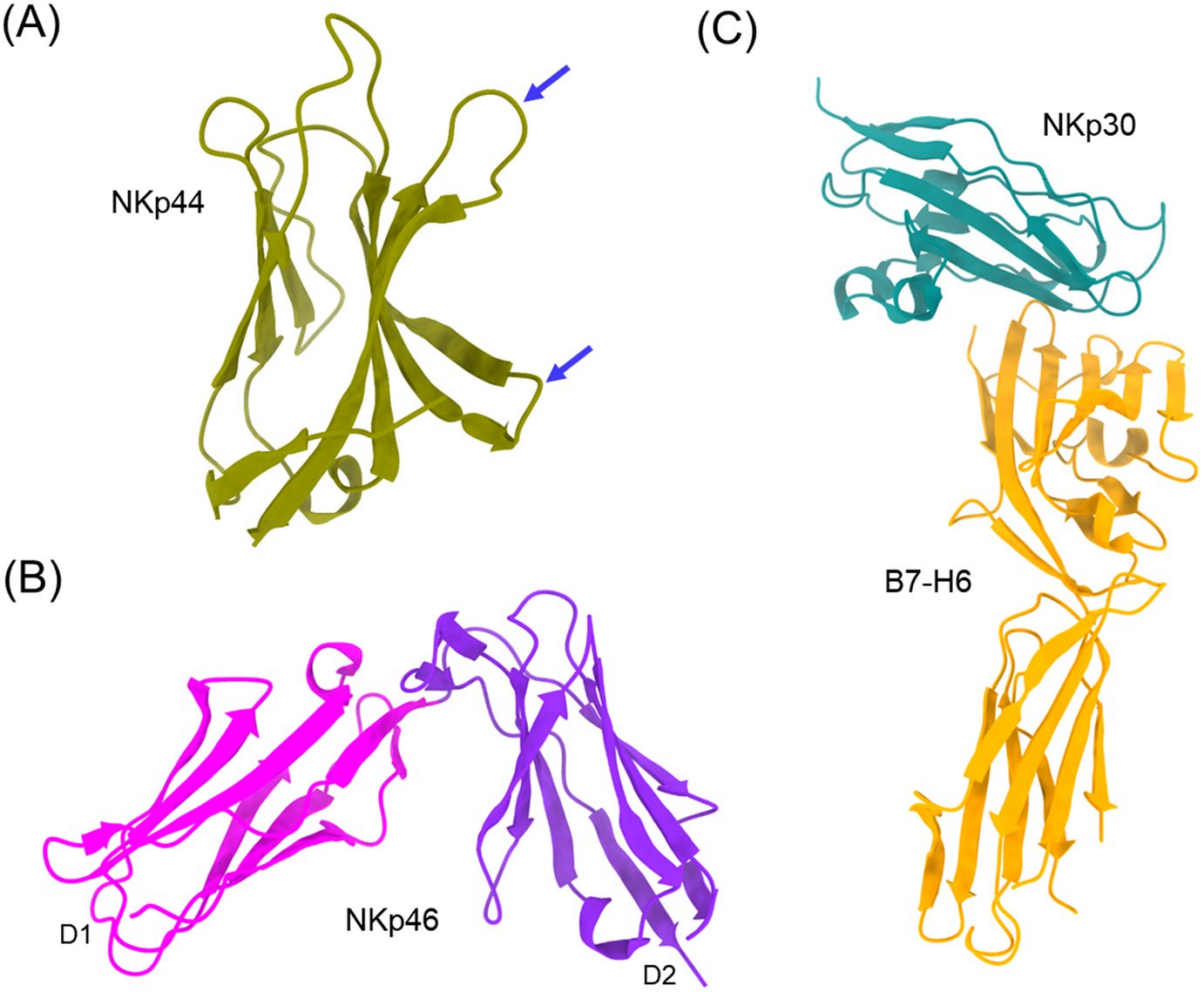
Structures of human natural cytotoxicity receptors. (A) Structure of NKp44 (1HKF) [16]. The blue arrows point to loops forming a positively charged groove that may bind anionic ligands. (B) Structure of NKp46 (1P6F) [18]. (C) Structure of NKp30 bound to B7‐H6 (3PV6) [20], which is selectively expressed on tumors.

To date, structures have been determined for NKp30, NKp44, and NKp46 in unbound form (Table 1) [14, 16, 18]. However, the only reported structure of an NCR in ligand‐bound form is that of NKp30 in complex with B7‐H6 [20]. NKp44 comprises a single V‐type Ig‐like domain whose main feature is an electropositive groove that may be a possible binding site for anionic ligands such as sialic acid (Figure 6A) [16]. NKp46 contains two C2‐set Ig‐like domains whose disposition resembles those of the D1D2 domains of KIRs and LILRs (Figure 6B) [18].

NKp30 is composed of a single C1‐set Ig‐like domain whose closest structural homolog is the V‐like domain of PDL1, a ligand for the inhibitory receptor PD1 [14, 20]. B7‐H6, like other B7 family members, consists of a membrane‐distal V‐like domain and a membrane‐proximal C‐like domain [20]. In the NKp30–B7‐H6 complex, the binding interface is formed by the front and back β‐sheets of the NKp30 C‐like domain juxtaposed against the front β‐sheet of the B7‐H6 V‐like domain (Figure 6C). B7‐H6 binds NKp30 in an antibody‐like manner, such that the FG loop of the B7‐H6, which corresponds to complementarity‐determining region (CDR) 3, forms a protrusion that fits snugly into a groove on NKp30 [20]. Additional interactions are mediated by the BC (CDR1‐like) and C'C" (CDR2‐like) loops of B7‐H6. How NKp30, a relatively small receptor, can recognize such structurally unrelated ligands as B7‐H6, BAT3 [88], HCMV pp65 [89], and β‐1,3‐glucan [90] is not apparent and must await structure determination of the corresponding complexes.

## 7 Recognition of MHC‐I Homologs by NKG2D

NKG2D is a homodimeric C‐type lectin‐like receptor found on NK cells and cytotoxic T cells. It recognizes several MHC‐I structural homologs, notably MICA, MICB, RAE‐1β, and ULBP1‐3, which all lack a peptide‐binding groove and β_2_m [28, 94]. In humans, MICA and MICB are upregulated in tumors compared to normal tissues [95, 96], whereas in rodents RAE‐1, H‐60, and MULT‐1 are upregulated [97, 98]. In humans, the HCMV‐encoded immunoevasin UL16 functions as a decoy receptor by binding MICB, ULBP1, and ULBP2 [99], thereby evading NKG2D‐mediated antiviral responses. In mice, the MCMV‐encoded immunoevasins m152, m155, and m145 downregulate the NKG2D ligands RAE‐1, H60, and MULT‐1, respectively. Crystal structures have been described for unbound human and mouse NKG2D [22, 100], for mouse NKG2D in complex with RAE‐1β [26], and for human NKG2D in complex with ULBP3 and MICA (Table 1) [24, 28]. Structures have also been determined of UL16 bound to MICB [99] and of m152 bound to RAE‐1β [101].

**FIGURE 7:**
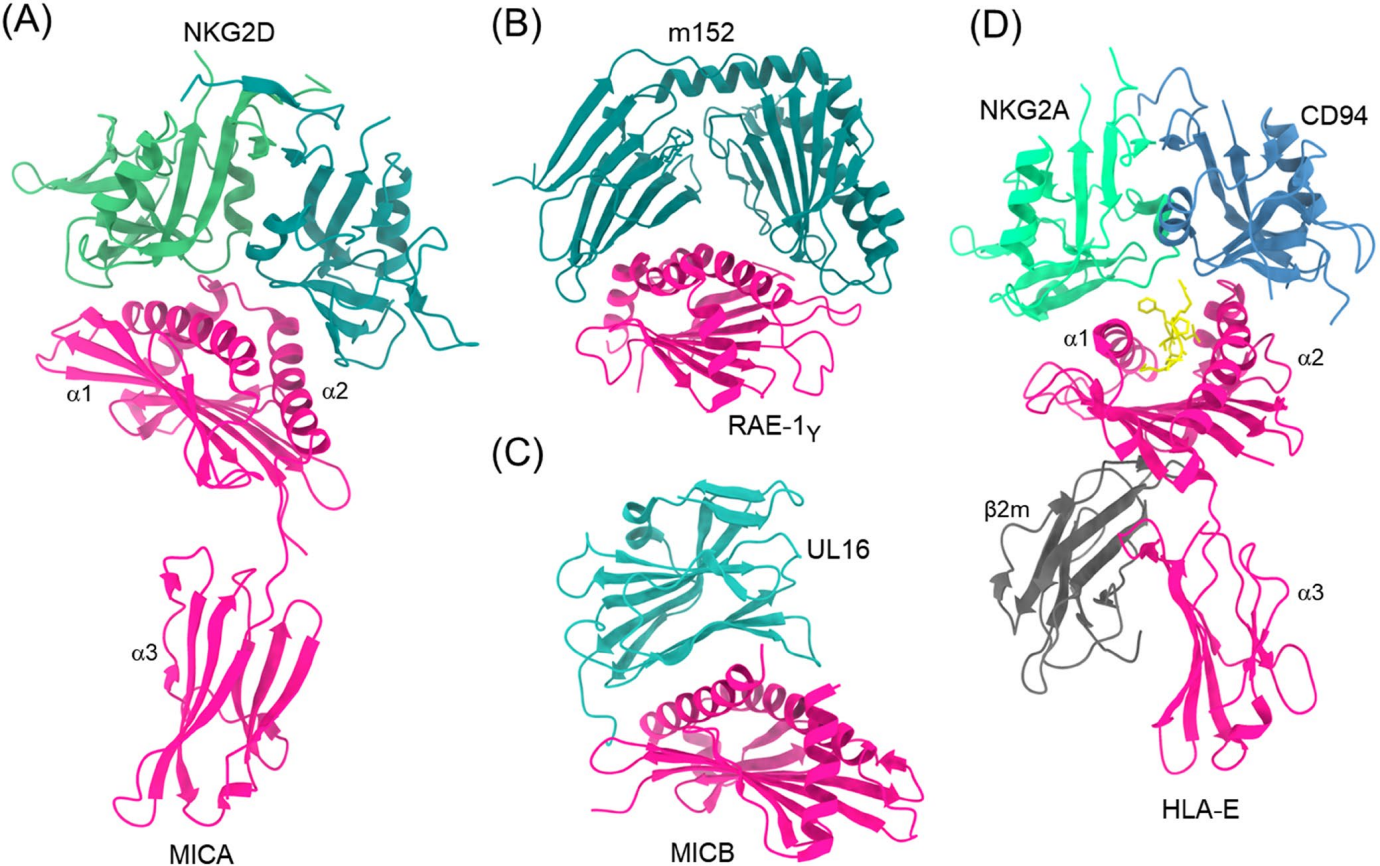
Structures of NKG2D and NKG2A/CD94 complexes. (A) Complex between NKG2D and MICA (1HYR) [24]. (B) Complex between the MCMV immunoevasin m157 NKG2D ligand RAE‐1γ (1JSK) [26]. (C) Structure of the HCMV immunoevasin UL16 bound to MICB (2WY3) [99]. (D) Structure of human NKG2A/CD94 bound to HLA‐E (3CDG) [30].

The MHC‐I homolog MICA contains an α1/α2 platform domain with α1 and α2 helices that form the peptide‐binding groove in *bona fide* MHC‐I molecules but that lacks peptide in MICA (Figure 7A) [102]. MICA includes an Ig‐like α3 domain but no β_2_m. In the NKG2D–MICA complex [24], the NKG2D homodimer binds orthogonally to the α1 and α2 helices of the platform domain in manner that roughly resembles that of KIRs on MHC‐I (Figure 3B) but that is completely distinct from the ways Ly49s and LILRs bind MHC‐I (Figures 1A,C and 5A). NKG2D engages ULBP3 and RAE‐1β [26, 28] with a docking topology similar to that observed in the NKG2D–MICA complex. The remarkable ability of NKG2D to use a single binding site to recognize ligands that share only ~25% sequence identity is explained by mutational analysis of NKG2D, which revealed that the most energetically important residues of NKG2D interact with conserved residues of MICA, ULBP3, and RAE‐1β [22, 103].

In the complex between the MHC‐like MCMV immunoevasin m152 and RAE‐1γ [101], m152 contacts the α1 and α2 helices of RAE‐1γ in pincer‐like fashion resembling the interaction between NKG2D and MICA (Figure 7B). In contrast, the Ig‐like HCMV immunoevasin UL16 employs a three‐stranded β‐sheet to bind the α1 and α2 helices of MICB (Figure 7C). UL16 and m152 promote viral survival by preventing NKG2D‐mediated NK cell activation through competition with NKG2D [104, 105, 106].

## 8 Recognition of HLA‐E by NKG2/CD94

Besides the homodimeric NKG2D receptor, the NKG2D family includes NKG2A, NKG2B, NKG2C, and NKG2E, which form homodimers with CD94 [107, 108, 109]. Whereas NKG2A and NKG2B are inhibitory receptors, NKG2C and NKG2E are activating. The ligand for NKG2D/CD94 receptors is HLA‐E. This non‐classical MHC‐I molecule binds peptides derived from the leader sequences of both classical and non‐classical MHC‐I molecules [107, 108, 109]. Expression of HLA‐E on cell surfaces depends on the production of other MHC‐I molecules because HLA‐E cannot be expressed without a bound peptide. Therefore, HLA‐E recognition by NKG2D/CD94 permits NK cells to detect expression of other MHC‐I molecules on target cells.

The structure of NKG2A/CD94 has been reported in unliganded form [110] and in complex with HLA‐E bound to a peptide from the HLA‐G leader sequence [30, 111]. The NKG2A and CD94 subunits contact the α1 and α2 helices of HLA‐E, respectively, such that NKG2A/CD94 straddles the peptide‐binding groove (Figure 7D). The invariant CD94 subunit, rather than NKG2A, dominates interactions with the peptide and is situated over the P8 residue. Of note, sequence variation among HLA‐E‐restricted peptides is concentrated in C‐terminal residues, which are read out by CD94 [30, 111].

## 9 NK Receptor Recognition of Cell–Cell Adhesion Proteins

Killer cell lectin‐like receptor G1 (KLRG1) and CD96 recognize the cell–cell adhesion proteins E‐cadherin and nectin‐like protein‐5 (necl‐5), respectively. KLRG1 is an inhibitory receptor expressed on most NK cells whose expression is highly upregulated following infection with viruses or parasites [112, 113, 114, 115, 116, 117]. E‐cadherin, its biological ligand, is located at the basolateral membrane of epithelial cells where it establishes tight binding between neighboring cells in adherens junctions. The interaction of KLRG1 with E‐cadherin prevents tissue damage by preventing epithelial cells expressing E‐cadherin from being lysed by KLRG1^+^ NK cells [118, 119, 120]. In addition, KLRG1 contributes to cancer immunosurveillance by detecting potentially metastatic epithelial tumors with downregulated E‐cadherin expression [120, 121, 122]. CD96 is an inhibitory receptor expressed on NK cells and CD8^+^ T cells that binds necl‐5 and is a potential target for cancer immunotherapy [123]. Necl‐5 is often upregulated on tumor cells and increased necl‐5 expression correlates with poor prognosis, possibly through inhibition of killing by CD96^+^ NK cells.

**FIGURE 8:**
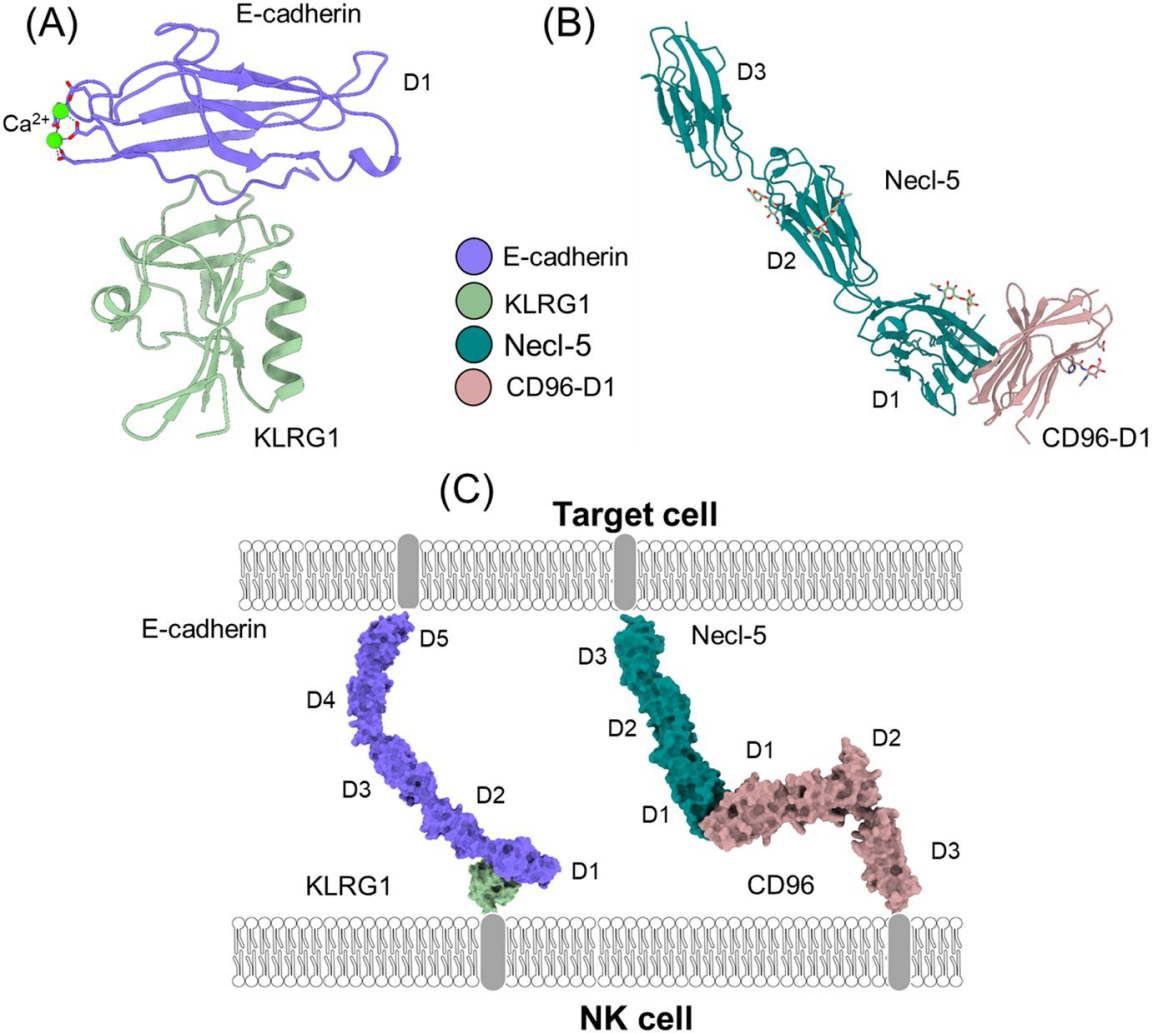
Recognition of cell–cell adhesion proteins by KLRG1 and CD96. (A) Structure of KLRG1 bound to the membrane‐distal D1 domain of E‐cadherin (3FF8) [34]. Bound Ca^2+^ ions are shown as green spheres. (B) Structure of the membrane‐distal D1 domain of CD96 bound to necl‐5 (6ARQ) [37]. *N*‐linked glycans are drawn in stick representation. (C) (left) Space‐filling model of the KLRG1–E‐cadherin complex. Ig‐like domains of E‐cadherin are labeled D1–D5. The model was constructed by superposing the structure of KLRG1 bound to D1 of E‐cadherin onto the structure of full‐length E‐cadherin (D1–D5) (3Q2V) [124]. (right) Space‐filling model of the CD96–necl‐5 complex. CD96 comprises three Ig‐like domains, but only D1 is present in the crystal structure. CD96 D2 and D3 were homology‐modeled using SWISS‐MODEL.

Crystal structures have been determined for KLRG1 bound to E‐cadherin [34] and for CD96 bound to necl‐5 (Table 1) [37]. KLRG1 is a type II transmembrane composed of a single extracellular C‐type lectin‐like domain (CTLD) connected by a stalk to transmembrane and cytoplasmic regions. The extracellular region of E‐cadherin comprises five Ig‐like domains (EC1–EC5). In the KLRG1–E‐cadherin complex, one KLRG1 CTLD binds one EC1 domain (Figure 8A) [34]. KLRG1 recognition of its non‐MHC ligand is reminiscent of Ly49C recognition of MHC‐I, where each CTLD of the Ly49C homodimer contains an entire ligand‐binding site (Figure 1C). By comparison, the binding site of NKG2A for MICA [24] is formed by two associated CTLD subunits (Figure 7A), as is the binding site of NKG2A/CD94 for HLA‐E (Figure 7D) [30, 111]. KLRG1 mainly contacts residues Val3‐Ile7 of E‐cadherin. These residues are completely conserved in N‐ and R‐cadherins, which are also KLRG1 ligands [34, 118]. This enables NK cells bearing a single KLRG1 receptor to monitor expression of multiple cadherins on target cells.

Whereas KLRG1 is a type II C‐type lectin‐like transmembrane protein, CD96, like its necl‐5 ligand, is a type I transmembrane protein comprising three extracellular Ig‐like domains [37]. The CD96–necl‐5 interaction involves only the first Ig‐like domain (D1) of each partner (Figure 8B). The overall docking topology resembles that observed for TIGIT, an NK inhibitory receptor closely related to CD96, in complex with nectin‐2 [125]. However. CD96 differs from TIGIT in terms of specificity. Whereas TIGIT recognizes nectin‐2, nectin‐3, and necl‐5, CD96 only binds necl‐5. The more restricted specificity of CD96 is attributable to a shift in CD96 D1 away from the CC" loop of necl‐5, leading to a loss of contacts with the FG loop of the receptor that stabilizes the TIGIT‐nectin‐2 complex [37]. Both KLRG1 and CD96 form elongated structures of ~190 Å at NK cell–target cell interfaces upon engaging their cell–cell adhesion protein ligands (Figure 8C).

## 10 Genetically Coupled C‐Type Lectin‐Like Receptor–Ligand Pairs

The NK gene complex (NKC) encodes approximately 30 type II transmembrane glycoproteins belonging to the C‐type lectin‐like superfamily [126]. NKC genes are classified into killer cell lectin‐like receptor (KLR) genes and C‐type lectin receptor (CLEC) genes. KLR genes encode proteins expressed on NK cells; CLEC genes encode proteins expressed on dendritic, myeloid, and other cell types. The KLR family of receptors includes Ly49, NKG2D, NKG2A/CD94, and KLRG1, which bind MHC‐I, MHC‐I‐like, or non‐MHC molecules (e.g., E‐cadherin), as described above. In addition, some KLR family members recognize CLEC2 proteins which themselves belong to the C‐type lectin‐like superfamily [127]. The genes encoding these KLR–CLEC2 receptor–ligand pairs, which modulate (increase or decrease) NK cell‐mediated cytotoxicity, are genetically linked in the NKC. These pairs include NKp65–KACL [127, 128, 129], NKp80–AICL [127, 130, 131], Nkrp1–Clr [132, 133], and NKR‐P1A–LLT1 [134, 135, 136, 137]. Crystal structures have been reported for human NKp65 bound to KACL [39], mouse NKR‐P1B bound to Clr‐b [42], and human NKR‐P1A bound to LLT1 (Table 1) [41].

**FIGURE 9:**
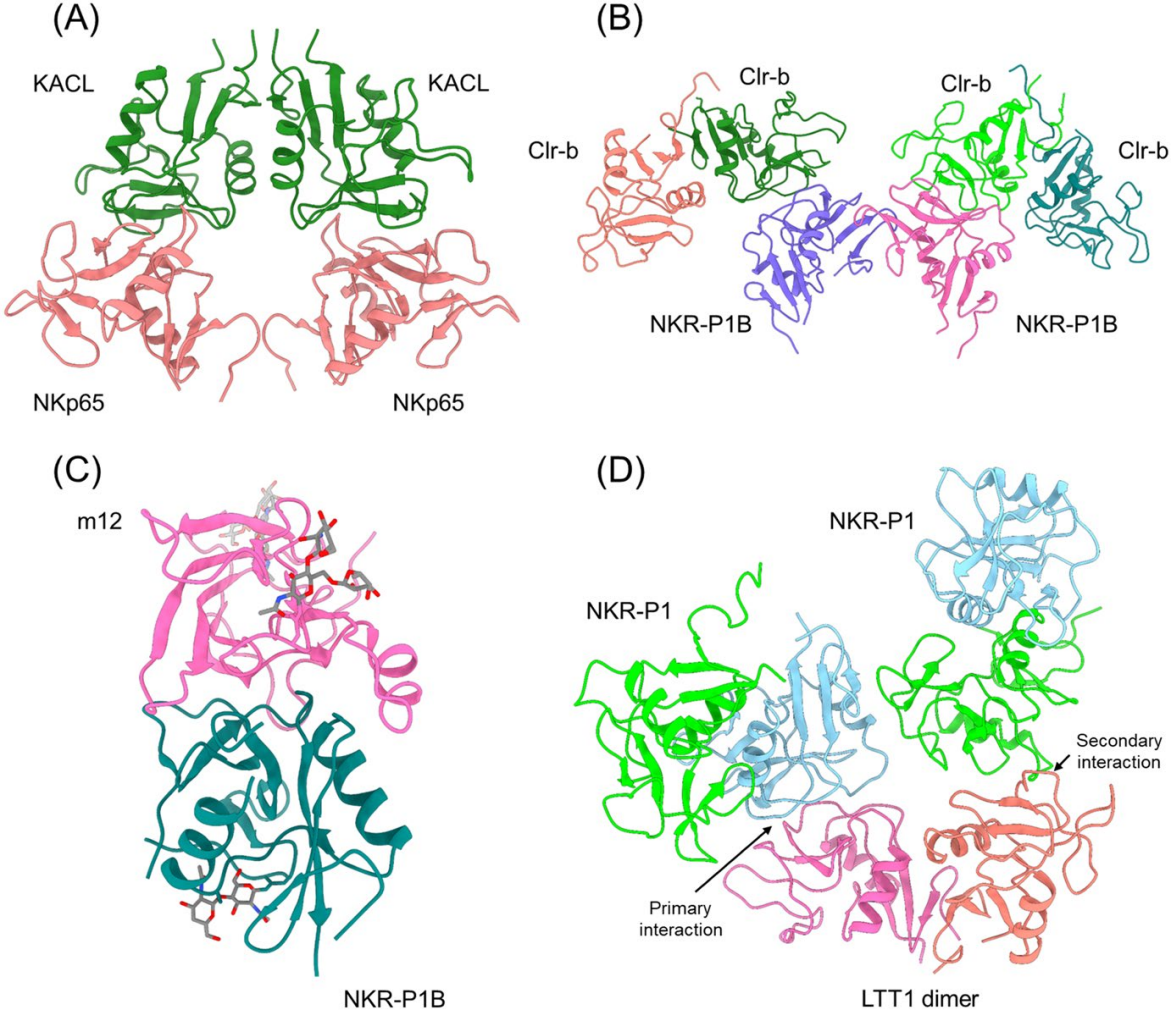
C‐type lectin‐like receptor–ligand pairs in the NK gene complex. (A) Structure of the human NKp65–KACL complex (4IOP) [39]. The NKp65 and KACL homodimers bind in a head‐to‐head orientation. (B) Structure of the mouse NKR‐P1B–Clr‐b complex (6E7D) [42]. Each Clr‐b dimer binds one monomer of the NKR‐P1B dimer. (C) Structure of the mouse NKR‐P1B–m12 complex (5TZN) [44]. *N*‐linked glycans are shown as sticks. The m12 immunoevasin straddles NKR‐P1B in a claw‐like manner. (D) Structure of the human NKR‐P1–LLT1 complex (5MGT) [41]. The LLT1 dimer (pink/orange) contacts the NKR‐P1 dimer formed by the green and light blue monomers. The light blue NKR‐P1 monomer binds LLT1 in the primary interaction mode. The green NKR‐P1 monomer binds LLT1 in the secondary interaction mode.

NKp65 stimulates NK cytotoxicity and release of proinflammatory cytokines upon binding KACL on keratinocytes [127, 128]. KACL is expressed almost exclusively in the skin, indicating that the NKp65–KACL pair plays a dedicated role in immunosurveillance of human skin. Whereas NKp65 is monomeric, KACL forms a homodimer similar to the Ly49 and NKG2D homodimers [39]. In the human NKp65–KACL complex, the two C‐type lectin‐like proteins engage each other in a head‐to‐head orientation (Figure 9A). The KACL homodimer binds NKp65 bivalently, with each KACL subunit constituting an independent binding site for NKp65 and each NKp65 monomer making identical interactions with KACL.

NKp65 binds KACL with remarkably high affinity (*K*_D_ = 6.7 × 10^−10^ M) compared to the micromolar *K*_D_s characteristic of other cell–cell recognition molecules [39]. This affinity is 3000‐fold greater than that of NKp80 for AICL [130] and 70,000‐fold greater than that of NKR‐P1A for LLT1 [138]. Unlike NKp80 and NKR‐P1A, which form disulfide‐linked dimers on the NK cell surface, NKp65 is not disulfide‐linked. Moreover, AICL and LLT1 [135, 136], but not KACL [128] exist as disulfide‐linked dimers on cells. Dimerization of NKp80 and NKR‐P1A may compensate for their low affinities compared to NKp65 by increasing avidity through bivalent binding to their ligands. The high affinity of NKp65 may circumvent the need for receptor dimerization by generating sufficiently stable complexes with KACL to drive efficient signaling.

NKR‐P1B is an inhibitory receptor that binds Clr‐b, which is considered a broad marker of healthy self. Downregulation of Clr‐b has been implicated in missing‐self recognition of virally infected and cancer cells [132, 133]. In the mouse NKR‐P1B–Clr‐b complex [42], two Clr‐b dimers interact head‐to‐head with one NKR‐P1B dimer positioned between them, such that each Clr‐b dimer binds one of the two NKR‐P1B subunits (Figure 9B). The docking mode in the NKR‐P1B–Clr‐b complex is similar to that in the NKp65–KACL complex (Figure 9A), suggesting that an evolutionarily conserved topology applies to other NKC‐encoded receptor–ligand pairs. The affinity of the NKR‐P1B–Clr‐b interaction in solution is extremely weak (*K*_D_ > 500 μM), likely due to poor shape complementarity at the interface [42]. This affinity may be insufficient to form stable receptor–ligand complexes to trigger signaling. Productive interactions may require additional avidity conferred by cross‐linking NKR‐P1B–Clr‐b complexes on the NK cell surface by the NKR‐P1B homodimer.

The structure of mouse NKR‐P1B bound to the MCMV‐encoded protein m12 has also been determined (Figure 9C) [44]. This immunoevasin restrains NK cell effector function by directly engaging the inhibitory NKR‐P1B receptor. Unlike Clr‐b, which possesses a C‐type lectin‐like domain (CTLD) fold, m12 adopts an eight‐stranded Ig‐like β‐sandwich fold comprising one large and one small β‐sheet. In the NKR‐P1B–m12 complex, m12 lies across the top of the NKR‐P1B CTLD, such that the m12 β‐sheets lie parallel to the interface (Figure 9C) [44]. The affinity of m12 for NKR‐P1B (*K*_D_ = 5.8 μM) far exceeds that of Clr‐b (*K*_D_ > 500 μM), which would enable m12 to easily outcompete Clr‐b for binding to the receptor.

NKR‐P1 is an inhibitory receptor that binds LLT1, which is mainly expressed on activated B cells and monocytes [139]. This interaction helps maintain NK cell self‐tolerance in these cells. LLT1 is upregulated on glioblastoma [136], B cell non‐Hodgkin's lymphoma [140], and other cancer cells, thereby contributing to immune evasion by attenuating NK cell cytotoxicity. In the human NKR‐P1–LLT1 complex, NKR‐P1 and LLT1 establish two types of contact (Figure 9D) [41]. The primary interaction mode closely matches that observed in the NKp65–KACL complex. However, the secondary interaction mode is unique to the NKR‐P1–LLT1 complex and involves a different region of LLT1 than the region used in the primary interaction mode. Both interaction modes position the membrane‐proximal parts of NKR‐P1 and LLT1 on opposite sides of the complex, creating a plausible model for interaction between two opposing cells [41]. In support of this model, ligation of both LLT1 binding interfaces was found to be required for effective NKR‐P1 inhibitory signaling.

## 11 Future Directions

The structural studies presented in this review have given us an atomic‐level understanding of how representative NK receptors recognize self and viral ligands, and thus discriminate between normal and tumor or virus‐infected cells. Despite these advances, the biophysical mechanisms by which inhibitory or activating signals are transmitted to the NK cell across the cell membrane remain largely unknown. It is also a mystery how NK cells integrate inhibitory and activating signals to determine the outcome of NK cell–target cell encounters. Research on membrane‐embedded NK receptors is required to obtain structural information linking ligand engagement to NK cell triggering. Such information cannot come from X‐ray crystallography of isolated NK receptor ectodomains, as described in this review, but may come from cryogenic electron microscopy (cryoEM) analysis of intact NK receptors and their associated DAP10, DAP12, or other signaling molecules, either in detergent or in the native‐like lipid bilayers of nanodics [141].

## Conflicts of Interest

The authors declare no conflicts of interest.

## Data Availability

Data sharing is not applicable to this article as no new data were created or analyzed in this study.
